# Chronic myelomonocytic leukemia as a cause of fatal uncontrolled inflammation in familial Mediterranean fever

**DOI:** 10.1186/1546-0096-13-S1-P177

**Published:** 2015-09-28

**Authors:** F Awad, S Georgin-Lavialle, A Brignier, C Derrieux, A Aouba, K Stankovic Stojanovic, G Grateau, S Amselem, S-A Karabina

**Affiliations:** 1 Hôpital Trousseau, Service de Génétique, Paris, France; 2 Sorbonne Universités, UPMC University Paris 06, INSERM UMR_S933, Paris, France; 3 Hôpital Tenon, Service de Médecine Interne, Paris, France; 4 Université Paris Descartes - Sorbonne Paris Cité, Service d'Hématologie Clinique, AP-HP, Hôpital Necker, Paris, France; 5 Hôpital Necker, Laboratoire d'Hématologie Biologique, Paris, France

## Introduction

Familial Mediterranean fever (FMF) is an autosomal recessive autoinflammatory disorder caused by mutations (mainly p.M694V in exon 10) in the *MEFV* gene. It is the most common hereditary fever syndrome. Daily and lifelong colchicine administration can prevent both fever attacks and occurrence of inflammatory amyloidosis. Chronic myelomonocytic leukemia (CMML) is a clonal hematopoietic stem cell disorder classified as a myelodysplastic/myeloproliferative neoplasm. The median age of CMML diagnosis is 70 years and current treatment includes hydroxyurea and/or 5-azacitidine.

## Objective

Circulating monocytes express the *MEFV* gene responsible for FMF. Monocytes are also the target cells of CMML. We aimed to test the inflammatory status of monocytes in a patient with a severe clinical phenotype combining FMF and CMML.

## Patients and methods

We report on an FMF patient who developed CMML leading to an uncontrolled and fatal inflammatory syndrome. Nine FMF patients, including the CMML patient, were included in this study. IL-1β, IL-6 and IL-18 cytokine levels were measured by ELISA in the plasma from patients and apparently healthy donors.

## Results

The patient, who was homozygous for the p.M694V mutation, was explored at the age of 83 for a profound anemia revealing a myelodysplastic syndrome. Despite colchicine therapy, an important inflammatory syndrome persisted. His status deteriorated quickly with severe uncontrolled inflammation and occurrence of peripheral monocytosis revealing the transformation of his myelodysplastic syndrome into CMML, with fatal outcome within a few months. Plasma levels of IL-6 and IL-18 were found to be very high, as compared to healthy controls and other CMML-free FMF patients.

## Conclusions

Our study unveils the interplay between two different disorders involving the same target cells, suggesting that in myelodysplasia with inflammatory manifestations, mutations in genes causing autoinflammatory syndromes, like *MEFV*, can be present and thus could be sought. Early chemotherapy with interleukin inhibitors could be proposed in such unusual situations.

